# Evaluation of the Bridge-in, Objective, Preassessment, Participatory Learning, Postassessment, and Summary–Based Instructional Model in Disaster Nursing: Quasi-Experimental Study

**DOI:** 10.2196/77728

**Published:** 2026-10-05

**Authors:** Li Pei, Taoyu Jia, Yaxin Liu, Xiao-li Pang, Muzi Li, Haoying Dou

**Affiliations:** 1 Tianjin University of Traditional Chinese Medicine, No.10 Poyang Lake Road, Tuanbo New City West, Jinghai District, Tianjin, 301617, China, 13820837386

**Keywords:** disaster nursing education, BOPPPS, competency, curriculum construction, undergraduate, Bridge-in, Objective, Preassessment, Participatory Learning, Postassessment, and Summary

## Abstract

**Background:**

The undergraduate stage is critical for developing nursing students’ disaster nursing competencies; however, traditional lecture-based teaching often fails to promote active learning, and innovative instructional models remain underused in this field. The BOPPPS (Bridge-in, Objective, Preassessment, Participatory Learning, Postassessment, and Summary) model is a student-centered, closed-loop instructional framework that emphasizes active participatory learning and real-time feedback.

**Objective:**

This study aimed to evaluate the effects of a BOPPPS-based blended teaching model on disaster nursing competencies and academic outcomes among undergraduate nursing students.

**Methods:**

This study used a quasi-experimental design with 77 undergraduate nursing students allocated to 3 groups: an experimental group receiving BOPPPS-based blended teaching (n=27), a control group with traditional lecture-based teaching (n=25), and a comparison group without systematic instruction (n=25). Questionnaires were used to assess students’ self-perceived disaster nursing knowledge, professional operational skills, and overall comprehensive competencies. The final examination assessed students’ mastery of theoretical knowledge and applied reasoning.

**Results:**

All 3 groups showed significant within-group improvements in disaster competence (all *P*<.001, n=77). Intergroup comparisons revealed significant differences (*H*=8.843, *P*=.01, η²=0.093); post hoc analysis showed that the experimental group (mean rank=48.79) scored significantly higher than the comparison group (mean rank=29.76, *P*_adj_=.009), but not significantly higher than the control group (mean rank=38.32, *P*_adj_=.34). The experimental group had a significantly higher overall course score than the control group (86.93, SD 5.08 vs 81.60, SD 6.90; *t*_50_*=*−3.186, *P*=.002).

**Conclusions:**

The implementation of a disaster nursing curriculum positively impacts students’ disaster nursing competency. Since the BOPPPS-based blended teaching model promotes active student engagement and improves teaching quality, we recommend its integration into disaster nursing education.

## Introduction

### Background

Disaster events present a significant threat to social development and human survival. Over recent years, there has been an increasing trend in the frequency of disasters worldwide [1]. Disasters can impose enormous stress on communities, leading to widespread disruption of daily life and challenging the resilience of social structures [2]. Disaster nursing is a crucial aspect of emergency relief, involving caregivers who use their specialized knowledge and disaster-related skills to provide urgent assistance to those affected by disasters. The primary goal is to minimize the adverse effects on public health and safety, reducing the damage caused by the disaster event [3]. Nurses are an essential component of the rescue team and play a crucial role in the overall functioning of the rescue system. They ensure that rescue operations run effectively while providing compassionate care to those in need. During and after disasters, nurses can critically assist patients, enhancing the quality of care and supporting recovery efforts [4]. The disaster nursing competencies of nurses directly impact the efficiency of disaster relief efforts [5]. Enhancing nurses’ disaster nursing skills enables them to respond swiftly and effectively during emergencies, thereby minimizing the societal losses caused by disasters. Research has demonstrated that nurses frequently experience heightened levels of physical and mental stress during periods of uncertainty and shock following disasters [6]. Although there is generally a high level of willingness among nursing students to participate in disaster relief, many are still reluctant to engage in rescue operations involving infectious diseases or man-made disasters [7]. The root cause of this reluctance among nursing students stems from complex psychological and structural factors, rather than a mere apprehension toward disaster events. Empirical evidence from our previous mixed methods inquiry regarding the baseline status indicates that students’ deployment willingness and response behaviors are profoundly shaped by their foundational disaster literacy, self-efficacy, and career adaptability [8]. Perceived deficits in disaster nursing competency can trigger situational anxiety when anticipating high-risk environments, thereby undermining students’ psychological readiness. Consequently, implementing targeted disaster nursing education that bridges the gap between theoretical knowledge and psychological adjustment is crucial to dismantle these cognitive and psychological barriers, thereby comprehensively enhancing students’ actual preparedness.

In China, disaster nursing education is still in the early stages of development and remains in its infancy, lacking a systematic and comprehensive academic framework as well as standardized teaching methodologies [9]. Consequently, only a limited number of colleges and universities currently offer specialized courses in this domain, with existing programs relying heavily on traditional lecture-based teaching. This conventional paradigm introduces severe pedagogical limitations, such as placing an overemphasis on book-based theoretical knowledge, unbalancing the relationship between teaching and learning, and marginalizing students’ central role in the learning process while suppressing their clinical initiative and creativity [10]. To overcome these pedagogical limitations, modern disaster education requires alternative active learning frameworks that reposition students as central participants. BOPPPS, a structured instructional design model, was introduced by education scholar Douglas Kerr in 1978 [11]. This model provides a framework for creating effective and engaging lessons by emphasizing 6 key components: Bridge-in (B), Objective (O), Preassessment (P), Participatory Learning (P), Postassessment (P), and Summary (S). Unlike traditional lecture-based teaching, this instructional approach provides teachers with a clearer framework for structuring lessons. By using preassessment and postassessment scores as timely feedback, educators can flexibly adjust their teaching strategies and master students’ learning progress. This teaching model emphasizes the creation of a student-centered learning environment, supporting students in managing their own learning content and providing flexible learning spaces that encourage purposeful engagement and demonstration of knowledge. This teaching model, which focuses on cultivating students’ curiosity, has proven effective in boosting learning satisfaction and significantly improving instructional efficacy [12]. Concurrently, research shows that early preparedness training during undergraduate study enhances the development of disaster nursing skills [13].

Over the past decade, various medical disciplines in China have gradually integrated the BOPPPS teaching model. Despite this widespread multidisciplinary adoption, empirical applications within the highly specialized field of disaster nursing remain largely absent from domestic research. The course is designed to enable students to (1) acquire core knowledge of disaster nursing, (2) understand the principles of emergency response and basic first aid, and (3) develop nursing competencies for common disaster scenarios. Accordingly, we conducted a quasi-experimental study to address the following research question: Is the BOPPPS-based blended teaching model more effective than traditional lecture-based teaching in improving undergraduate nursing students’ disaster nursing competency and academic performance? We hypothesized that, compared with the control group, the experimental group would achieve significantly greater gains in disaster nursing competency and significantly higher scores on the objective final examination. The findings of this study are intended to inform nursing educators, disaster nursing curriculum designers, and clinical emergency training managers seeking to strengthen course design and instructional strategies in disaster nursing education.

### Conceptual Framework

Drawing on the BOPPPS model, we constructed a blended teaching framework for disaster nursing education that comprises 4 interrelated components (Figure 1). Component 1 (standardization with personalization) establishes an integrated online-offline teaching structure in which content, resources, and assessment standards are unified and classroom-based real-time feedback enables differentiated guidance. Component 2 (student-centered pedagogy) activates students’ intrinsic motivation through clear learning objectives and participatory learning contexts, thereby shifting them from passive reception to active inquiry. Component 3 (technological support) uses the Chaoxing Learning Platform to integrate online resources, in-person lectures, and after-class interaction, enhancing learning efficiency. Component 4 (nursing talent development) follows three guiding principles: consolidating the knowledge foundation, fostering systematic and critical thinking, and enhancing knowledge transfer ability. This conceptual framework guided the subsequent instructional design and outcome evaluation of the course.

**Figure 1.**
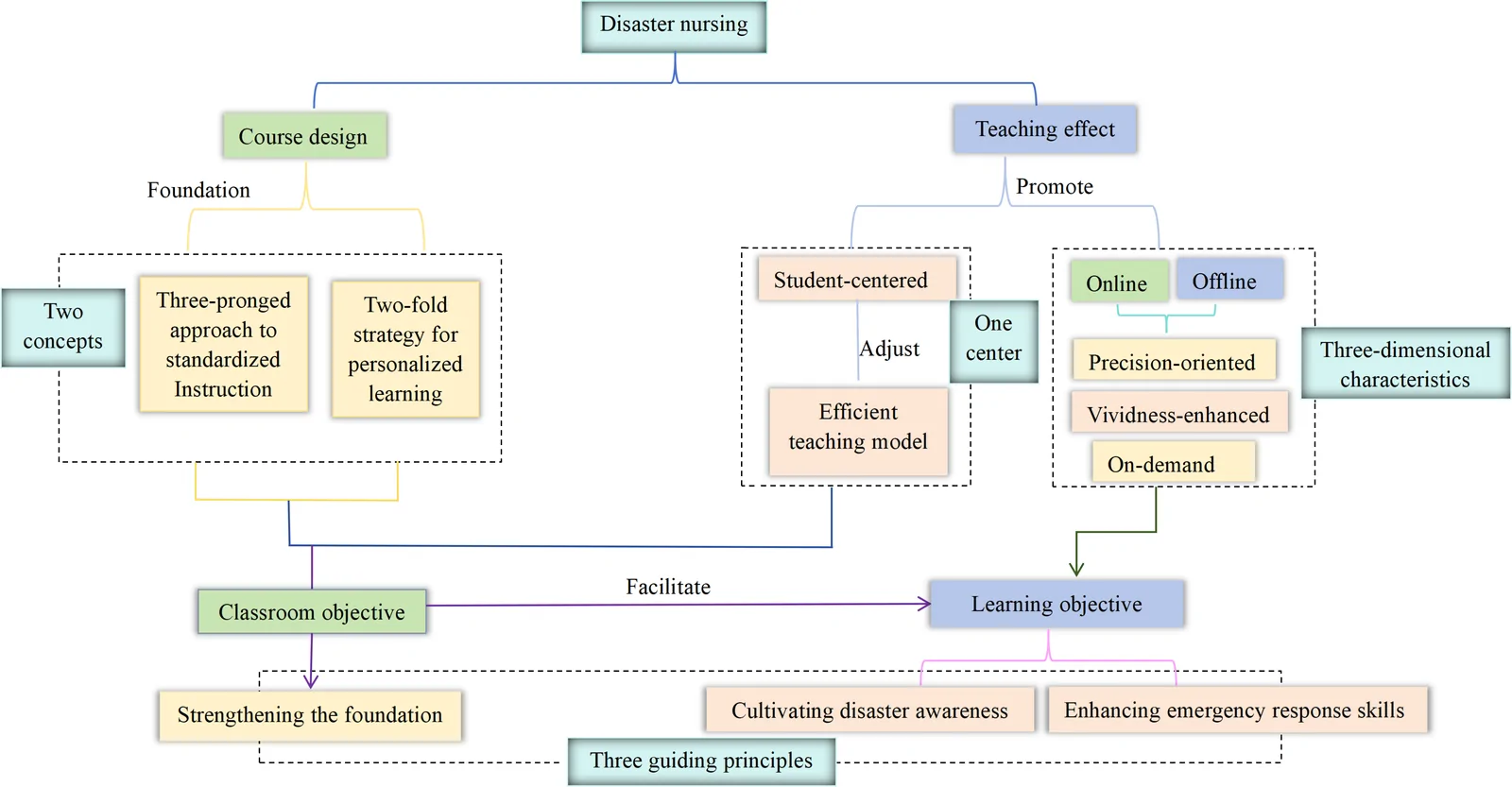
The conceptual framework of curriculum construction.

## Methods

### Study Design

We conducted this study during the second semester of the 2023 to 2024 academic year. To implement the study effectively, we systematically developed syllabi, instructional designs, evaluation methods, and quality control measures. We developed the curriculum design and instructional modules of the disaster nursing course based on the International Council of Nurses (ICN) Core Competencies in Disaster Nursing Version 2.0, jointly issued by the ICN and the World Health Organization (WHO) [14]. To bridge the gap between these macro standards and undergraduate requirements, our research group used a 2-round Delphi expert consultation to systematically select and adapt the specific course topics, learning objectives, and competencies. We convened a panel of 15 multidisciplinary experts, all of whom had more than 10 years of professional experience and intermediate or higher titles. Through this consensus-building process, we refined the initial 5 primary and 14 secondary domains based on expert feedback from the first round, which optimized specific item phrasings (eg, modifying “first-aid skills” to “on-site rescue skills”) and added critical dimensions, such as rehabilitation for postdisaster psychological issues, while explicitly considering cross-disciplinary integration with other nursing tracks. Consequently, this rigorous adaptation resulted in a curriculum with a total of 27 instructional hours comprising 9 hours of online instruction and 18 hours of face-to-face instruction, organized into 5 teaching units and 13 content items, as shown in Table 1.

**Table 1.** Course framework and clock hours.

Teaching unit and content	Face-to-face instruction	Online instruction	Total hours
Chapter 1: Introduction			
Concept and classification of disasters	2	—^a^	2
Disaster emergency response system	0.5	1	1.5
Management of disaster stages	0.5	—	0.5
Chapter 2: Common disaster rescue and care			
Natural disaster nursing	4	2	6
Nursing in technological disasters	3	3	6
Nursing care for common medical conditions in disaster rescue	3	—	3
Basic on-site disaster rescue skills	—	3	3
Chapter 3: Psychological intervention in disaster rescue			
Overview, psychological characteristics, and interventions for affected populations	0.5	—	0.5
Mental health maintenance and intervention for rescue workers	0.5	—	0.5
Chapter 4: Postdisaster rehabilitation and nursing			
Rehabilitation of common neurological disorders after disasters	1	—	1
Rehabilitation of common musculoskeletal disorders after disasters	1	—	1
Chapter 5: Hygiene and epidemic prevention in disaster rescue			
Postdisaster environmental health protection	1	—	1
Population health protection	1	—	1
Total	18	9	27

a Not applicable.

This study included 3 groups. We determined group assignments based on the students’ voluntary enrollment in the elective course sessions. Participants registered for the Tuesday class comprised the experimental group and received BOPPPS-based blended teaching. Those enrolled in the Monday session formed the control group and received traditional lecture-based teaching. Additionally, we used convenience sampling to recruit a comparison group from students in the same academic cohort who did not enroll in the elective. Participants in this group did not receive any disaster nursing education, and their data served as a baseline for determining whether the specialized curriculum was the sole driver of improvements in students’ disaster response capabilities. Both the control and experimental groups attended the course in parallel and completed all assigned learning tasks; Table 2 details the specific curricular differences. To ensure the comparability of baseline levels across groups, all 3 cohorts participated in an identical core nursing curriculum, with the “Disaster Nursing” elective course serving as the sole independent variable. During the intervention, we administered precourse and postcourse questionnaires to all groups, assessing self-perceived disaster nursing knowledge, professional operational skills, and overall comprehensive competencies to verify the intervention’s efficacy. At the end of the semester, students in the experimental and control groups completed a unified, comprehensive final examination assessing theoretical knowledge and applied reasoning, thereby comparing the BOPPPS-based blended teaching with traditional lecture-based teaching.

**Table 2.** Comparison of instructional activities and design.

Phase	BOPPPS^a^-based blended teaching (experimental group, n=27)	Traditional lecture–based teaching (control group, n=25)	Overlap (shared elements)
Preclass	Bridge-in, Objective, Preassessment: Students previewed courseware, reviewed learning objectives, and completed diagnostic quizzes via the Chaoxing platform.	General autonomous preview without structured guidance or digital tracking.	Same textbook and preparatory topics, completed the same preassessment.
In-class (1)	Participatory Learning: Students engaged in active learning through classroom presentations and group discussions.	Teacher-led lecturing with Q&A sessions.	Same curriculum standards and core knowledge points.
In-class (2)	Postassessment: Real-time knowledge testing using the interactive “Discussion” function of the Chaoxing platform to monitor mastery.	Standardized posttest conducted at the end of the session to assess immediate mastery.	Both groups completed the same posttest content for outcome evaluation.
In-class (3)	Summary: Structured conclusion by teachers based on real-time feedback and knowledge mapping.	Teacher-led summary of key points from the lecture.	Same concluding knowledge framework.
Postclass	Consolidation: Knowledge reinforcement through Chaoxing resources integrated into the consolidation stage of BOPPPS, with structured review.	Knowledge review via personal notes using the same Chaoxing resources.	Same reference materials and study goals.

a BOPPPS: Bridge-In, Objective, Preassessment, Participatory Learning, Postassessment, and Summary.

The control group primarily used the traditional lecture-based teaching, with the following structured steps: in the precourse phase, teachers clarified the teaching objectives by outlining the essential textbook knowledge and skills that students needed to master. They meticulously planned the teaching content to ensure alignment with these objectives, selected and collected relevant case studies and supplementary materials to support learning, and designed appropriate teaching methods to effectively deliver the curriculum. Second, during in-class instruction, the teacher explained the theoretical knowledge from the textbook, supplemented by PowerPoint slides that included videos related to disaster nursing and case studies to enhance understanding. Students were encouraged to outline key points from the textbook content and take detailed notes during the lectures. The teacher then guided students’ thinking through the analysis of provided cases, fostering active participation in group discussions and responding to teacher-led questions. Finally, the teacher summarized the key points covered in the class and addressed any questions raised by the students, ensuring that all concepts were clearly understood.

The experimental group delivered the course based on the BOPPPS teaching model, incorporating course resources from the Chaoxing Learning Platform. This teaching model comprised 3 main phases: preclass preparation, implementation during the lesson, and consolidation and feedback at the end of the lesson. The 6 core components of the BOPPPS model, including Bridge-in, Objective, Preassessment, Participatory Learning, Postassessment, and Summary, span both the preclass and in-class phases. This structure enabled students to focus and systematize their knowledge effectively. The preclass preparation stage: (1) Bridge-in: Teachers distributed introductory videos and instructional materials related to the disaster nursing course to students via the Chaoxing Learning Platform, designed to present knowledge content more intuitively and engagingly, thereby deepening students’ cognitive impressions and enhancing the quality of preclass preparation. (2) Objective: The teacher clearly defined the teaching objectives and delivered the guided-learning content to students via the online platform. Students then completed the preclass guided learning tasks on the Chaoxing Learning Platform, establishing clear learning expectations before class, thereby making in-class activities more targeted and effective. (3) Preassessment: Students completed the corresponding preassessment questions based on the teaching materials released by the teacher, thereby clarifying their current level of knowledge mastery and identifying weak points to focus on in their subsequent learning. The in-class implementation phase: (4) Participatory Learning: Teachers systematically reviewed and analyzed the key content of newly introduced courses, facilitating teacher-student interactions through the Chaoxing Learning Platform. This included posing questions on critical knowledge points and encouraging students to engage in in-depth discussions and rapid responses to relevant queries. To elevate student engagement, we designed a series of in-class participatory activities, including group presentations and case analyses, fostering an interactive learning environment. (5) Postassessment: During the postassessment phase, teachers posted follow-up test questions and facilitated Q&A sessions to clarify any remaining doubts. Additionally, they guided students in summarizing their reflections via the Chaoxing Learning Platform. Students submitted their quiz answers on time during class, provided constructive feedback on their learning experiences, and posed questions following submission. (6) Summary: Upon completion of the teaching of key content and related classroom activities, the teacher provided a comprehensive summary of the material covered, highlighting key points and clarifying any challenging areas. Postcourse consolidation and feedback phase: After class, students used the course materials available on the Chaoxing Learning Platform to consolidate and reinforce what had been covered during the lesson. The specific teaching process of the BOPPPS-based blended curriculum construction is shown in Figure 2.

**Figure 2.**
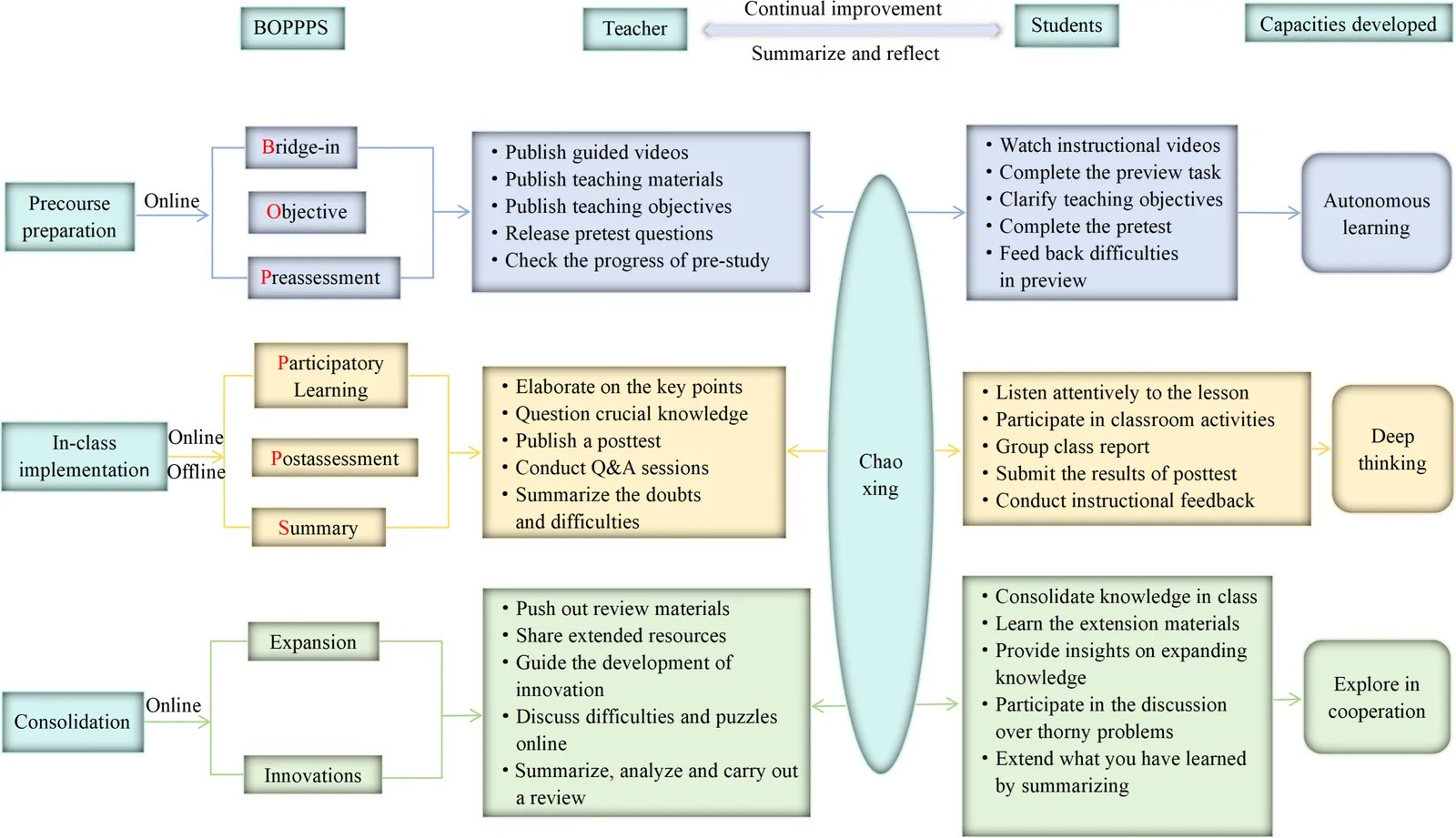
Flowchart of Chaoxing Learning Platform-Bridge-In, Objective, Preassessment, Participatory Learning, Postassessment, and Summary (BOPPPS) blended instructional design.

### Course Implementation Context

At the beginning of each semester, the university’s Academic Affairs Office publishes an official administrative document regarding the optimization of the undergraduate elective course system, thereby formally activating the application and development process for elective courses across the entire university. To align with the practical requirements of course development, our research team first guided students in implementing a multimedia project aimed at promoting disaster nursing knowledge, while conducting a preliminary needs assessment to evaluate nursing students’ baseline disaster competencies and learning willingness. Researchers regularly compiled student feedback and, in conjunction with the assessment results, dynamically optimized the elective course content within the university’s overarching curriculum framework, thereby providing empirical evidence to support the design and approval of the course. In accordance with the standards outlined in the regulatory documents, our team submitted a comprehensive course proposal to the academic administration, detailing the course rationale, learning objectives, curriculum content, and implementation plan. The department responsible for the course established an independent evaluation panel comprising at least 3 senior academic experts holding associate professor titles or higher, alongside invited external peer experts to ensure optimal review objectivity. Using uniform quality assessment standards, this review panel conducted a comprehensive evaluation of the course across multiple critical dimensions, including the feasibility of the learning objectives, the appropriateness of the class schedule, and the structural fidelity of the syllabus. Curricula securing a comprehensive score of 80 or higher were eligible for development approval upon successful validation. Having met the required standard, the disaster nursing course was formally included in the undergraduate talent cultivation scheme and officially commenced during the second semester of the 2023 to 2024 academic year.

### Participants

This study recruited 77 third-year undergraduate nursing students from Tianjin University of Traditional Chinese Medicine, employing a quasi-experimental design. We recruited undergraduate nursing students prior to the commencement of the academic semester, using an open-enrollment framework to offer the disaster nursing course as an elective to all third-year nursing students. The inclusion criteria were as follows: (1) full-time undergraduate nursing students; (2) no prior experience with disaster nursing courses that integrated the traditional curriculum; and (3) voluntary participation in this study and signing of the informed consent form. To implement the recruitment process, the research team promoted information about the elective course through the university’s channels, and students enrolled voluntarily based on their learning interests.

### Questionnaire Survey

#### General Information Questionnaire

The research team developed a self-administered general information questionnaire to collect participants’ general information, including home location (county/town vs city), whether they voluntarily chose the nursing program, whether they had been taught disaster prevention knowledge and skills by their parents, and whether they had attended relevant practical training.

#### Disaster Nursing Competency Scale

To evaluate the course’s effectiveness, we adopted a dual approach combining questionnaires and a final examination. For the questionnaire component, all 77 participants completed precourse and postcourse tests via the Questionnaire Star platform, yielding a 100% effective response rate. This study employed the Disaster Nursing Competency Scale (DNCS) developed by Hu [15] to assess participants’ disaster nursing competencies. This instrument is theoretically grounded in the core-competency framework for disaster nursing jointly established by the ICN and the WHO, and was originally developed using a mixed methods approach involving a literature review, 2 rounds of Delphi expert consultations, and a pilot survey. The scale comprises three first-level indicators, 11 second-level indicators, and 50 third-level indicators. The first-level indicators include knowledge system, professional competence, and comprehensive qualities. The second-level indicators include disaster nursing foundational knowledge, disaster nursing specialized knowledge, assessment and observation skills, nonemergency response capabilities, on-site emergency care skills, disaster nursing management abilities, psychological crisis intervention skills, communication and collaboration abilities, physical fitness, psychological resilience, and moral and ethical standards. In the original development study, the scale demonstrated sound validity and reliability: the expert-rated Scale-level Content Validity Index was 0.95, correlation coefficients between first- and second-order dimensions ranged from 0.810 to 0.982 and from 0.394 to 0.976, respectively, and the Cronbach α and split-half reliability of the full scale reached 0.984 and 0.920, respectively. We used a 5-point Likert scale for all responses, with 1 indicating “very poor” and 5 indicating “very good”; total scores range from 50 to 250, with higher scores indicating greater disaster nursing competency. We used the original version of the scale directly, without any adjustments or omissions of any items, to preserve its structural integrity; in the current sample, the scale also demonstrated high internal consistency, with a Cronbach α of 0.927.

### Academic Achievement Assessment

To comprehensively evaluate students’ academic achievement, this study established a multidimensional course assessment framework. This framework derived the final course grade from a process assessment (40%) and a final examination (60%). The process assessment, accounting for 40% of the total course grade, was derived from objective data automatically collected through the university’s online learning platform. The process assessment comprised 5 weighted modules, with the combined weights totaling 100%: (1) chapter task points (25%). This module assessed the completion of video- and audio-based learning tasks. Students who completed all task points received full credit; otherwise, the score was calculated in proportion to the completed task points. (2) Chapter quizzes (20%). We administered chapter quizzes during each online class session to assess mastery of the session content. The module score was calculated as the average percentage across all chapter quizzes. (3) Group problem-based learning tasks (40%). Each group assignment was combined as a weighted average of instructor evaluation (60%), intergroup peer evaluation (10%), intragroup peer evaluation (20%), and self-evaluation by group members (10%). The instructor’s evaluation criteria included the appropriateness of the presentation materials, content accuracy, clarity of organization and logical rigor, quality of PowerPoint design, and the presenter’s delivery and demeanor. Each assignment was scored on a 100-point scale, and the module score was the average of all group assignment scores. (4) Attendance (10%). This module captured the attendance rate, defined as the number of sessions attended divided by the total number of required sessions. (5) Class discussions (5%). This module tracked points earned by students: 20 points for initiating a new topic, 5 points for replying to a topic, and 5 points for receiving a “like,” with a maximum total of 100 points. We scored all 5 modules on a 0 to 100 scale and aggregated them according to the weights above to produce the process assessment score. We developed the final examination for the disaster nursing course based on the course syllabus, which was structured according to the ICN/WHO disaster nursing core competency framework. To clarify the content basis of this objective academic assessment, the research team analyzed the assessment domains covered by the final examination. The intended learning objectives of the course were for students to be able to: (1) describe the concepts, classification, characteristics, and management phases of disasters and the structure of disaster emergency rescue systems; (2) apply nursing principles in common disaster rescue scenarios; (3) demonstrate knowledge of essential first-aid and emergency response skills in disaster settings; (4) identify major postdisaster nursing and rehabilitation needs; and (5) recognize common psychological responses after disasters and identify basic principles of psychological crisis intervention, and explain key principles of postdisaster public health protection and epidemic prevention. Because several learning objectives overlapped conceptually, the final examination was not designed with a strict one-to-one correspondence between each item and a single learning objective. Instead, it was structured and analyzed according to 5 assessment domains, each corresponding to one or more learning objectives. These domains included foundations of disaster nursing, common disaster rescue nursing and first aid, psychological intervention in disaster rescue, postdisaster rehabilitation and nursing, and hygiene and epidemic prevention in disaster rescue.

At the conclusion of the curriculum, we administered the summative evaluation through a standardized, invigilated offline final examination. The instructional emphasis of the course syllabus dictated the score distribution. We designed the 100-point assessment instrument to evaluate multiple cognitive domains. The detailed arrangement of question types and score distribution is as follows: (1) 25 single-choice questions (50 points), which primarily assessed students’ mastery of foundational theoretical knowledge across the major course modules, including disaster concepts and classification, disaster rescue principles, common disaster rescue nursing, psychological intervention, and postdisaster public health and epidemic prevention; (2) 15 true or false items (30 points), which mainly evaluated rapid judgment and recognition of correct actions in disaster-related situations, including on-site first aid, emergency response, safety principles, and postdisaster infection prevention measures; and (3) a case-based essay question (20 points), which tested higher-order reasoning and decision-making by requiring students to analyze a complex disaster scenario, identify key problems, and propose appropriate rescue and nursing interventions. To ensure methodological rigor and objective scoring, the final examination was administered under strict proctoring in a controlled examination environment. After the final examination, one senior instructor graded all responses according to a predefined, standardized scoring rubric to maintain scoring consistency. Upon completion of grading, the scores were entered into the course-assessment database for automatic calculation of the overall course score. The resulting final overall course score was then released to students via the university’s encrypted digital learning management system.

### Statistical Analysis

This study performed data entry and analysis using SPSS statistical software (version 27.0; IBM Corp). Categorical variables were summarized as frequency counts (n) and percentages (%), with the chi-square test evaluating between-group differences. The Shapiro-Wilk test assessed the normality of continuous variables. For normally distributed data, we reported variables as means (SDs), and used the independent-samples *t* test to compare the 2 groups. For nonnormally distributed data, we expressed variables as median (IQR) and applied the Mann-Whitney *U* test for 2 groups. Within-group pretest versus posttest paired comparisons were performed using the Wilcoxon signed-rank test, with effect size *r* calculated as *r*=|*Z*|/$\sqrt{n}$. According to Cohen [16] criteria, *r* values of 0.1, 0.3, and 0.5 indicate small, medium, and large effects, respectively. For comparisons among 3 groups, the Kruskal-Wallis *H* test compared the distributions, which were delineated using mean ranks. When the *H* test revealed a significant overall difference, Dunn post hoc test with Bonferroni correction was used for the pairwise comparisons. Furthermore, ordinal eta-squared (η^2^) captured the effect size via the formula η^2^=(*H*−*k*+1)/(*N*−*k*). Based on Cohen conventional benchmarks, η^2^ values of 0.01, 0.06, and 0.14 denote small, medium, and large effects, respectively [17]. Two-tailed *P*<.05 or *P*_adj_<.05 for post hoc analyses defined statistical significance.

### Ethical Considerations

This study obtained ethical clearance from the Medical Ethics Committee of Tianjin University of Traditional Chinese Medicine. The study involved the collection and analysis of data from the teaching intervention, student questionnaires, and course assessments. All participants provided informed consent before completing the questionnaire. The researchers also clarified to all participants that their course assessment scores would be used solely for data analysis within the scope of their informed consent. To ensure academic fairness, participants were explicitly informed that participation in or withdrawal from the survey would have no impact on their academic evaluations or final grades.

## Results

### Comparison of General Information Among the 3 Groups

Table 3 presents the baseline characteristics of the 77 participants by cohort. The 3 cohorts exhibited no significant differences in terms of home location, whether they chose the nursing program of their own volition, or whether they had attended practical training in disaster prevention skills. Among baseline characteristics, only parental teaching of disaster prevention knowledge and skills differed significantly across groups (*P*=.04). This variable was treated as a descriptive baseline characteristic and was not included as a covariate in subsequent analyses.

**Table 3.** Comparison of general information for undergraduate nursing students (N=77).

Variable	Experimental group (n=27), n (%)	Control group (n=25), n (%)	Comparison group (n=25), n (%)	Chi-square (*df*)	*P* value
Home location				1.299 (2)	.52
Counties and towns	15 (56)	16 (64)	12 (48)		
City	12 (44)	9 (36)	13 (52)		
Do you choose your nursing program by yourself?				4.069 (2)	.13
Yes	14 (52)	13 (52)	19 (76)		
No	13 (48)	12 (48)	6 (24)		
Have you been taught disaster prevention knowledge and skills by your parents?				6.432 (2)	.04
Yes	10 (37)	14 (56)	18 (72)		
No	17 (63)	11 (44)	7 (28)		
Have you attended any practical training on disaster prevention skills?				4.069 (2)	.13
Yes	14 (52)	19 (76)	13 (52)		
No	13 (48)	6 (24)	12 (48)		

### Comparison of Precourse and Postcourse Disaster Nursing Competency Scores and Score Gains Across the 3 Groups

As shown in Table 4, the results showed that undergraduate nursing students’ level of disaster nursing competency improved after the course implementation. On the postassessment, the experimental group scored higher than the control group (experimental: 210.00, IQR 194.00‐242.00 vs control: 200.00, IQR 186.50‐210.50). Wilcoxon signed-rank tests revealed significant within-group differences across all groups: control and comparison groups (*z*=−4.37, *r*=0.874, *P*<.001) and experimental group (*z*=−4.54, *r*=0.874, *P*<.001).

**Table 4.** Comparison of the disaster nursing competency scores before and after the educational intervention (N=77).

Group	Students, n	Pretest, median (IQR)	Posttest, median (IQR)	*z*	*r*	*P* value
Control group (didactic instruction)	25	148.00 (134.00‐165.00)	200.00 (186.50‐210.50)	−4.37	0.874	<.001
Comparison group (no instruction)	25	155.00 (143.00‐176.00)	200.00 (181.00‐214.00)	−4.37	0.874	<.001
Experimental group (BOPPPS^a^ model)	27	138.00 (127.00‐152.00)	210.00 (194.00‐242.00)	−4.54	0.874	<.001

a BOPPPS: Bridge-In, Objective, Preassessment, Participatory Learning, Postassessment, and Summary.

Table 5 presents the medians (IQRs) of disaster nursing competency gain scores (posttest − pretest) for the 3 groups, along with the between-group comparisons of these values. The Kruskal-Wallis *H* test showed a statistically significant difference among the 3 groups (*H*=8.843, *P*=.01, η²=0.093). Dunn post hoc test with Bonferroni correction showed that the experimental group (mean rank=48.79) scored significantly higher than the comparison group (mean rank=29.76, *z*=2.968, *P*_adj_=.009), but no statistically significant difference was observed between the experimental and control groups (mean rank=38.32, *z*=−1.589, *P*_adj_=.34), nor between the control group and the comparison group (*z*=1.353, *P*_adj_=.53).

**Table 5.** Comparison of the disaster nursing competency gain scores among the 3 groups (N=77).

	Students, n	Median (IQR)	Mean rank	*P* value	*H* test	η²
Group				.01	8.843	0.093
Control group (didactic instruction)	25	53.00 (36.50‐69.50)	38.32			
Comparison group (no instruction)	25	40.00 (25.00‐62.00)	29.76			
Experimental group (BOPPPS^a^ model)	27	61.00 (49.00‐86.00)	48.79			

a BOPPPS: Bridge-In, Objective, Preassessment, Participatory Learning, Postassessment, and Summary.

### Comparison of Academic Achievement Outcomes Between the Experimental and Control Groups

As shown in Table 6, the BOPPPS-based blended model produced significantly higher overall course scores in the experimental group (mean 86.93, SD 5.08) than in the control group (mean 81.60, SD 6.90; *t*_50_*=*−3.186, *P*=.002). Grade distributions shifted toward higher bands in the experimental group, with more students scoring in the 90 to 99 and 80 to 89 ranges and fewer in the 70 to 79 range, while the number of nonpassing scores remained comparable across groups (Figure 3). For the process assessment section, the score was higher in the experimental group (91.60, IQR 86.60‐94.00 vs 88.20, IQR 82.85‐90.65; *z*=1.969, *P*=.049). On the final examination, the experimental group also scored significantly higher than the control group (88.00, IQR 82.00‐92.00 vs 80.00, IQR 72.00‐87.00; *z*=3.107, *P*=.002). Further analysis of the exam submodule data revealed that the score advantage of the experimental group was mainly concentrated in the single-choice questions section, in which the experimental group scored significantly higher than the control group (42.00, IQR 40.00‐46.00 vs 40.00, IQR 34.00‐42.00; *z*=2.101, *P*=.04). By contrast, the true or false and case-based essay sections showed no significant differences (true/false: *z*=1.235, *P*=.22; case-based essay: *z*=0.739, *P*=.46).

**Table 6.** Comparison of course assessment components between the experimental and control groups (N=52).

Assessment component and group	Students, n	Median (IQR)	Mean (SD)	*t* test (*df*)/*z*	*P* value
Process assessment				1.969	.049
Control group	25	88.20 (82.85‐90.65)	—^a^		
Experimental group	27	91.60 (86.60‐94.00)	—		
Final examination				3.107	.002
Control group	25	80.00 (72.00‐87.00)	—		
Experimental group	27	88.00 (82.00‐92.00)	—		
Single-choice questions				2.101	.04
Control group	25	40.00 (34.00‐42.00)	—		
Experimental group	27	42.00 (40.00‐46.00)	—		
True/false questions				1.235	.22
Control group	25	26.00 (26.00‐29.00)	—		
Experimental group	27	28.00 (26.00‐28.00)	—		
Case-based essay question				0.739	.46
Control group	25	18.00 (5.00‐19.00)	—		
Experimental group	27	18.00 (18.00‐18.00)	—		
Overall course score				−3.186 (50)	.002
Control group	25	—	81.60 (6.90)		
Experimental group	27	—	86.93 (5.08)		

a Not applicable.

**Figure 3.**
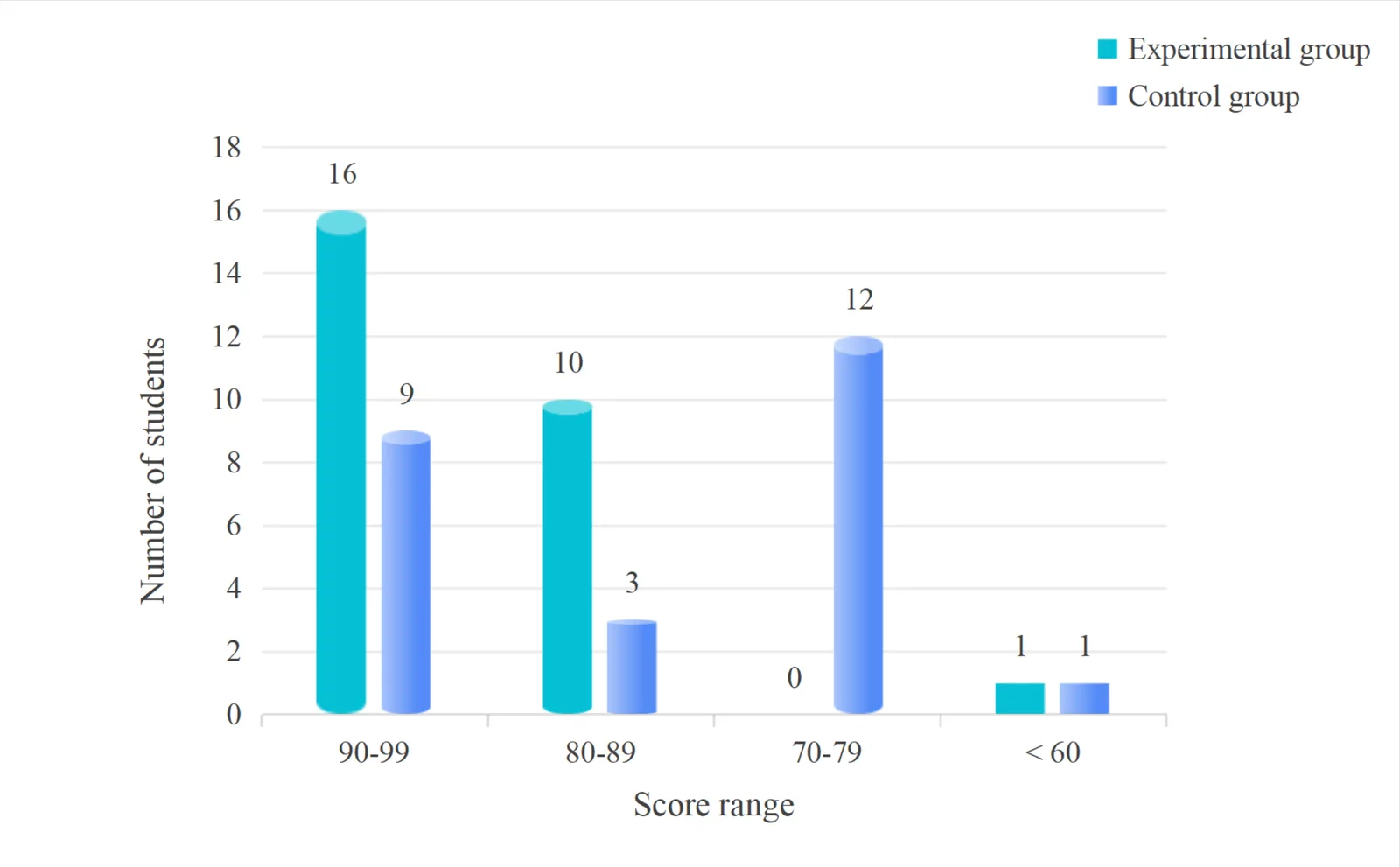
Overall course scores by group.

We analyzed the distribution of final examination items across assessment domains based on the course syllabus and the primary content assessed by each item. The final examination covered 5 assessment domains, each corresponding to one or more course learning objectives. Common disaster rescue nursing and first aid accounted for the largest proportion of the total score, followed by foundations of disaster nursing, hygiene and epidemic prevention in disaster rescue, psychological intervention in disaster rescue, and postdisaster rehabilitation and nursing. Postdisaster rehabilitation and nursing was assessed mainly through the second subquestion of the case-based essay item and represented a relatively small proportion of the total score. Table 7 presents the distribution of question formats and scores across assessment domains.

**Table 7.** Distribution of final examination items across assessment domains and related learning objectives.

Assessment domain	Related learning objectives	Single-choice questions	True/false questions	Case-based essay question	Score
Foundations of disaster nursing	Objective 1	4, 13, 14, 15, 16, 25	28, 29	N/A^a^	16
Common disaster rescue nursing and first aid	Objectives 2 and 3	1, 2, 5, 6, 7, 8, 9, 11, 12, 17, 24	30, 31, 32, 33, 34, 35, 36, 37, 38, 39	41(1)	52
Psychological intervention in disaster rescue	Objective 5	18, 19, 20, 22	26	N/A	10
Postdisaster rehabilitation and nursing	Objective 4	N/A	N/A	41(2)	10
Hygiene and epidemic prevention in disaster rescue	Objective 5	3, 10, 21, 23	27, 40	N/A	12
Total	Objectives 1‐5	25 items	15 items	1 item	100

a N/A: not applicable.

## Discussion

### Principal Findings

In this study, we employed a quasi-experimental design to analyze quantitative data to evaluate the effectiveness of the BOPPPS-based blended teaching model. The study yielded 3 principal findings. First, regarding the disaster nursing competency, the experimental group demonstrated greater improvement in competency scores than the control and comparison groups. Second, in the final examination, the experimental group achieved significantly higher total scores than the control group, primarily in the single-choice section. Third, the experimental group scored significantly higher in overall course score than the control group. These results partially confirm the hypothesis: the BOPPPS-based blended teaching is more effective than traditional lecture-based teaching or no systematic instruction in improving disaster nursing competency and objective academic outcomes.

### Implications of Findings

These findings offer several implications. First, the BOPPPS-based blended teaching model positively affected both students’ self-assessed competence and objective course grades, suggesting that it enhances not only perceived ability but also quantifiable learning outcomes, which supports its broader adoption in disaster nursing courses. Second, the Bridge-in and Preassessment components helped students enter a learning mindset early. This integration of online self-study with in-person instruction addresses a key limitation of traditional lecture-based teaching in promoting active learning. Third, because the experimental and control groups did not differ significantly in true/false questions or case-based essay questions, future courses should incorporate more simulation-based training, situational judgment tasks, and case analysis to support the development of higher-order applied competencies.

### Comparison to the Literature

Compared with previous studies, the present study addresses several limitations identified in earlier work through its structured design. Tussing et al [18] synthesized the literature on disaster preparedness education for nurses and nursing students and recommended digitalized teaching approaches; however, as a narrative review, their conclusions relied largely on inferential synthesis of previously published literature and lacked primary empirical data. The present study addresses this gap by providing quantitative evidence of the effectiveness of the BOPPPS-based blended teaching model in disaster nursing. Prior studies have demonstrated that high-immersion simulation approaches, such as virtual reality, can enhance disaster preparedness among nursing students [19], but these approaches typically require substantial hardware investment, and instructors cannot readily observe students’ operational details during simulation, which hampers timely feedback. In contrast, the BOPPPS-based blended teaching model implemented in this study relies on routine online learning resources and incorporates a structured real-time classroom feedback loop, offering a cost-effective alternative for disaster nursing education. Phan et al [20] conducted a low-cost disaster simulation exercise but collected data only after the intervention, which limited the ability to attribute score improvements to the teaching method. The present study addressed this by including a control group and employing a pretest-posttest design, allowing both within-group and between-group comparisons and providing stronger quantitative evidence that the observed score gains can be attributed to the teaching method. Glauberman et al [21] reported the DAIS tabletop exercise primarily through qualitative description and experience summary, without quantitative comparative evidence. It should be noted that DAIS focuses on interprofessional team collaboration, whereas the present study focuses on intraprofessional competency development through the BOPPPS-based blended teaching model. The findings of the present study can therefore serve as a foundation for single-discipline disaster nursing curricula and provide a basis for future designs that incorporate interprofessional collaboration. Accordingly, in a context where systematic disaster nursing education frameworks remain underdeveloped in China [9], the model proposed in this study represents a highly scalable and effective implementation strategy.

### Effects on Disaster Nursing Competency

Analysis of the DNCS scores indicated significant within-group improvements among all participants, suggesting that different forms of learning may help enhance students’ disaster nursing competency. Subsequent intergroup comparisons revealed significant differences in disaster nursing competency between the 3 groups. In terms of competency score gains, the experimental group showed the highest mean rank (48.79), followed by the control group (38.32) and the comparison group (29.76). This pattern suggests that the experimental group had the highest overall ranking in changes in disaster nursing competency. Dunn post hoc test showed a significant difference between the experimental group and the comparison group (*P*_adj_=.009), whereas the differences between the experimental and control groups (*P*_adj_=.34) and between the control and comparison groups did not reach statistical significance. These findings suggest that systematic teaching intervention may play an important role in improving disaster nursing competency, and that the BOPPPS-based blended teaching model showed a clearer beneficial effect than no systematic training. Although the experimental group had a higher mean rank than the control group, the difference between these 2 groups did not reach statistical significance. Therefore, this study cannot confirm a clear statistical advantage of the BOPPPS-based blended teaching over traditional lecture-based teaching. Future studies with larger sample sizes and greater statistical power should further verify this trend. Notably, although the control group had a higher mean rank than the comparison group, this difference also did not reach statistical significance. This finding suggests that only relying on traditional lecture-based teaching or relying on students’ existing professional experience may be insufficient to fully promote disaster nursing competency. Cho and Kwon [22] similarly reported that traditional disaster nursing education may not adequately develop students’ practical abilities and has limitations in maintaining long-term learning effects and improving students’ responses to nonroutine disaster situations. Therefore, future disaster nursing education should further strengthen structured, interactive, and scenario-based teaching designs to support the integrated development of students’ disaster nursing knowledge, skills, and clinical reasoning.

Furthermore, a significant difference was observed between the experimental and comparison groups but not between the experimental and control groups. This finding may be partly attributable to methodological limitations. First, the study may have lacked sufficient statistical power to detect subtle differences in pedagogical outcomes between the two intervention models. The experimental and control groups included only 27 and 25 participants, respectively. Although the overall effect size was moderate (η*²*=0.093), the achieved statistical power remained limited for detecting a medium-sized between-group effect. Larger multicenter studies are therefore needed to determine whether the trend toward higher scores in the BOPPPS teaching group represents a modest but genuine pedagogical advantage. Second, the DNCS is a subjective self-assessment instrument, and its sensitivity may be insufficient to capture changes in behavioral performance or subtle differences in skill mastery between two advanced teaching models. To address this measurement constraint, our protocol incorporated objective final examinations in addition to subjective self-assessment scales. Although the self-assessment scale did not reveal significant between-group differences, the objective final examination showed significantly higher scores in the BOPPPS teaching group than in the control group. This finding suggests that the BOPPPS model may be more effective in fostering classroom participation and deepening students’ understanding of the course content. This finding is consistent with the results reported by Dai et al [23], confirming that the BOPPPS-based blended teaching model yields more significant educational outcomes in nursing courses compared to traditional teaching methods and effectively improves students’ academic performance. Therefore, implementing specialized disaster nursing courses constitutes a pivotal professional empowerment initiative, equipping future frontline nurses with the essential technical capabilities required to respond to large-scale public health emergencies. This course not only fulfills nursing students’ interest in learning about this specialized field but also significantly improves their integrated mastery of disaster theory and practice through systematic training. Research indicates that robust disaster competence and knowledge foster a heightened willingness to engage in relevant activities and assume corresponding responsibilities [4,24]. Consequently, the competency gains achieved through the BOPPPS-based blended teaching model not only fortify nursing students’ professional qualifications but also instill the confidence necessary for active engagement and effective performance in real-world disaster response scenarios.

A notable finding requiring explanation is the improvement observed in the comparison group, which received no formal disaster nursing education; we offer several possible interpretations. First, an inherent selection bias and baseline imbalance heavily influenced student trajectories. Specifically, the comparison group demonstrated the highest baseline scores on the pretest (155 points) compared to the experimental (138 points) and control groups (148 points). This difference indicates that students who did not take the course already possessed a stronger foundation of existing knowledge, and their higher initial learning ability may have made it easier for them to achieve further self-directed improvement over time. Second, a test effect or learning effect may have emerged: participants completed the DNCS during the pretest, and this prior contact may have influenced the comparison group students’ awareness of disaster nursing concepts, thereby prompting them to engage in informal, self-directed learning. Third, intergroup interference inevitably blurred the boundaries of the intervention. Contamination may have occurred through informal peer discussions, as all 3 groups were drawn from the same academic cohort and shared common spaces (eg, dormitories, clinical placement sites). Comparison group students may have discussed disaster nursing topics with peers in the instructed groups, partially attenuating the observed differences between groups. Fourth, after a full semester of study, the natural accumulation and maturation of students’ overall clinical knowledge would also lead to a slight increase in their self-assessed professional competence. The study design did not include procedures to monitor and avoid such intergroup interference, which is a limitation of this study. Future research could address this issue by conducting cluster randomization at the institutional level or by staggering the study periods for different intervention groups. These considerations further highlight the necessity of using objective assessment metrics, such as final exams, in conjunction with self-assessment scales when evaluating teaching effectiveness.

### Effects on Academic Achievement Outcomes

The results of this study indicate that the experimental group, which adopted the BOPPPS-based blended teaching model, significantly outperformed the control group in both process assessment and final exam scores. Figure 3 further demonstrates that the experimental group had a higher proportion of students scoring in the excellent (90-99) and good (80-89) ranges for their overall course score. In contrast, fewer students in the experimental group fell into the intermediate score range (70-79), while the number of nonpassing scores was comparable between both groups. The results of this study align with previous research, indicating that blended teaching models have more positive effects compared to single traditional teaching methods [11]. This is because the BOPPPS-based blended teaching model excels by dividing the teaching and learning process into six distinct components: Bridge-in, Objective, Preassessment, Participatory Learning, Postassessment, and Summary. In this study, these components were organized into 3 phases: before, during, and after the lesson. This structured approach provides a clear learning path, significantly enhances students’ initiative, and helps them maintain better focus [25]. Studies have demonstrated that simulated mixed reality exercises enhance nursing students’ knowledge and understanding of real disaster scenarios. Notably, participatory learning, which is an indispensable component of this process, plays a crucial role in developing students’ practical problem-solving abilities [26,27]. Within the BOPPPS model, participatory learning effectively integrates theoretical knowledge and skills, promoting a student-centered approach to education [28].

Regarding process assessment, there was only a marginally significant difference between the 2 groups. This result demonstrates the rigorous process management employed in this study, with both groups maintaining consistency in behavioral compliance, including classroom attendance, completion of daily assignments, and participation in online learning. Therefore, the final examination score can serve as an objective core metric reflecting differences in students’ mastery of knowledge under different instructional interventions. In terms of the achievement of instructional content and objectives, this study found that the BOPPPS intervention was effective in helping students master basic disaster concepts, classifications, and rescue systems (Objective 1); nursing principles applied in common disaster scenarios such as fires and mudslides (Objective 2); the mastery of basic first aid and self-rescue skills, such as handling spinal fractures and treating burns (Objective 3); and postdisaster psychological disorders and the implementation of public health and epidemic prevention principles (Objective 5). However, the current assessment tools included relatively few items addressing Learning Objective 4, which focused on long-term postdisaster care and rehabilitation needs. As a result, the observed differences across question formats and total final examination scores may not be sufficient to determine whether the two groups differed in their deeper understanding of rehabilitation nursing. This limitation suggests that future course assessments should refine the distribution and weighting of items across learning objectives and include more diverse approaches, such as case-based essay questions, to ensure more comprehensive coverage of all intended learning outcomes. The results of this study show that the experimental group’s scores were significantly higher than those of the control group. Within specific sections, the experimental group’s advantage was primarily concentrated in the single-choice questions section, while no significant differences were observed in the true or false questions and the case-based essay questions. This pattern of an advantage that manifests selectively in single-choice items is consistent with the broader measurement evidence reported by Mee et al [29], who systematically analyzed question difficulty, discrimination, and response latency between objective choice questions and short-answer formats in medical student examinations. Their evaluation substantiated the conclusion that choice items can robustly segregate students based on their actual proficiency levels. The single-choice questions (totaling 50 points) in this exam are designed to comprehensively cover the course’s learning objectives, with content extending to specific core topics such as the international triage standards, first aid principles for sudden drowning and food poisoning, and public health measures following earthquakes and floods. In the BOPPPS-based blended teaching, teachers facilitated diverse activities such as classroom presentations and group discussions. These interactive processes enabled students to exchange ideas and share opinions, fostering critical thinking and teamwork skills. By leveraging students’ individual strengths, these activities enhanced the interactivity of the teaching process and established effective communication and feedback mechanisms between teachers and students, as well as among students. The integration of an immediate feedback mechanism into instruction effectively facilitates students’ mastery of foundational knowledge across diverse domains and systematically promotes the construction of an integrated theoretical framework [30]. Consequently, students in the experimental group scored significantly higher on single-choice questions (*P*=.04), and this improvement contributes to the achievement of core instructional objectives. The true or false questions primarily assessed students’ rapid recognition of appropriate emergency response principles and procedures in disaster scenarios, whereas the case-based essay question emphasized the integration of clinical presentations, triage priorities, emergency interventions, and postdisaster nursing needs. These findings suggest that the effect of short-term BOPPPS-based blended teaching on rapid situational judgment and higher-order case-based reasoning remains to be further examined. Compared with basic knowledge recognition, these abilities depend more heavily on situational experience, sequential decision-making practice, exposure to complex scenarios, and feedback on clinical decision-making. This interpretation is consistent with previous systematic review evidence indicating that simulation-based instruction can support the development of clinical reasoning in undergraduate nursing students [31]. Therefore, classroom-based structural optimization alone may not be sufficient to produce clear between-group differences within a short course period. Future course designs should consider extending instructional hours and incorporating more in-depth case discussions, scenario-based simulation training, and situational judgment tasks to better foster students’ higher-order competencies. In summary, the BOPPPS-based blended teaching markedly promotes students’ capacity to construct and retain core theoretical knowledge across multiple modules, thereby advancing the achievement of Objectives 1, 2, 3, and 5 through enhanced structured engagement. In medical education, the BOPPPS model has been effectively utilized across multiple disciplines, proving to be a highly effective teaching framework. It promotes sustainable development in students’ future careers and aligns with the current trend in medical education, which emphasizes ability cultivation and practical application.

### Limitations

This study has several limitations. First, the sample comprised only 77 students from a single institution, which may limit the generalizability of the findings. Second, a few baseline characteristics differed significantly between groups. Although the primary outcomes were analyzed using nonparametric tests without covariate adjustment, residual confounding cannot be fully excluded. Third, the study’s one-semester timeframe did not permit the evaluation of long-term knowledge retention or the application of skills in professional settings. Fourth, our recruitment strategy introduced a self-selection bias because the disaster nursing curriculum was administered as an elective course. Fifth, the final examination was newly developed for this study and has not been validated; its content and construct validity remain to be established. Finally, despite efforts to maintain group separation, the quasi-experimental design within a single institution cannot fully eliminate the possibility of inter-group contamination. Future research should therefore prioritize multi-institutional, longitudinal investigations with larger sample sizes that account for baseline balancing and covariate adjustment, validate the examination through expert review and repeated administration, and integrate simulated disaster scenarios to evaluate the BOPPPS model’s long-term impact on students’ clinical competencies and professional growth.

### Conclusions

This study compared the effectiveness of a blended teaching model based on the BOPPPS framework with traditional teaching methods in disaster nursing education for undergraduate students. It investigated the impact of the course curriculum on students’ disaster nursing competency. The results indicated that students taught using the BOPPPS-based blended teaching model demonstrated a significantly higher overall course score than those who received traditional lecture-based teaching. The disaster nursing curriculum was found to be effective in improving students’ disaster response skills. As an innovative teaching model, BOPPPS effectively engages students’ initiative, enhances their interest in learning, and develops essential professional skills and emergency response capabilities for disaster scenarios. Future studies could further validate the effectiveness and applicability of BOPPPS by integrating various teaching modes and utilizing advanced educational technologies. This would not only provide valuable insights for enhancing disaster nursing education at other Chinese universities but also establish a strong foundation for cultivating nursing professionals with advanced disaster management competencies.

## Abbreviations

BOPPPS: Bridge-in, Objective, Preassessment, Participatory Learning, Postassessment, and Summary
DNCS: Disaster Nursing Competency Scale
ICN: International Council of Nurses
WHO: World Health Organization

## References

[1] Stimpson JP, Rashed AL, Pandya J, Baudot EC, Whitfill J, Ortega AN (2025). Health equity in the wake of disasters and extreme weather: evidence from an umbrella review. Health Aff Sch.

[2] Han W, Liang C, Jiang B, Ma W, Zhang Y (2016). Major natural disasters in China, 1985-2014: occurrence and damages. Int J Environ Res Public Health.

[3] Zhang D, Zhang L, Gong A (2021). Development of disaster nursing in China: from the spirit of Nightingale to COVID-19. Disaster Med Public Health Prep.

[4] Taskiran G, Baykal U (2019). Nurses’ disaster preparedness and core competencies in Turkey: a descriptive correlational design. Int Nurs Rev.

[5] van Esch A, Daehnert E, Bramer WM, Gommers D, van Mol MMC (2026). Disaster preparedness and needed competencies among critical care nurses: a scoping review. BMC Nurs.

[6] Nekooei Moghaddam M, Saeed S, Khanjani N, Arab M (2014). Nurses’ requirements for relief and casualty support in disasters: a qualitative study. Nurs Midwifery Stud.

[7] Tayebi Z, Norouzinia R, Moatadelro Z, Pour AF, Nourian B (2024). Nursing students’ willingness to respond in disasters: a cross sectional study of facilitators and barriers. BMC Nurs.

[8] Zhou M, Pei L (2025). Research on status and influencing factors of disaster literacy among medical students based on mixed methods [Article in Chinese]. Health Vocational Education.

[9] Zhang YY, Zhu LL, Sheng Y, Li XH, Xu XH, Wang QY (2018). Disaster nursing development in China and other countries: a bibliometric study. J Nurs Scholarsh.

[10] Ma X, Zeng D, Wang J, Xu K, Li L (2022). Effectiveness of bridge-in, objective, pre-assessment, participatory learning, post-assessment, and summary teaching strategy in Chinese medical education: a systematic review and meta-analysis. Front Med (Lausanne).

[11] Hu K, Ma RJ, Ma C, Zheng QK, Sun ZG (2022). Comparison of the BOPPPS model and traditional instructional approaches in thoracic surgery education. BMC Med Educ.

[12] Kılıç N, Şimşek N (2019). The effects of psychological first aid training on disaster preparedness perception and self-efficacy. Nurse Educ Today.

[13] Li Z, Cai X, Zhou K (2023). Effects of BOPPPS combined with TBL in surgical nursing for nursing undergraduates: a mixed-method study. BMC Nurs.

[14] (2019). Core competencies in disaster nursing: version 2.0. https://www.icn.ch/resources/publications-and-reports/core-competencies-disaster-nursing-version-20.

[15] Hu X (2020). Research on competency-based disaster nursing training curriculum setting [Article in Chinese] [Master’s thesis].

[16] Cohen J (1988). Statistical Power Analysis for the Behavioral Sciences.

[17] Fiel Peres F (2026). Effect sizes for nonparametric tests. Biochem Med (Zagreb).

[18] Tussing TE, Chesnick H, Jackson A (2022). Disaster preparedness: keeping nursing staff and students at the ready. Nurs Clin North Am.

[19] Shujuan L, Mawpin T, Meichan C, Weijun X, Jing W, Biru L (2022). The use of virtual reality to improve disaster preparedness among nursing students: a randomized study. J Nurs Educ.

[20] Phan Q, Geller DE, Broughton AS, Swan BA, Wells JS (2023). Evaluating a low-cost disaster preparedness simulation for prelicensure nursing students. Disaster Med Public Health Prep.

[21] Glauberman GHR, Wong LCK, Bray ML, Katz AR (2020). Disaster aftermath interprofessional simulation: promoting nursing students’ preparedness for interprofessional teamwork. J Nurs Educ.

[22] Cho M, Kwon M (2025). Sustainable disaster nursing education through functional exercises and simulation: effects on knowledge, problem-solving, and learning outcomes. Sustainability.

[23] Dai Y, He Q, Lei L (2026). Effect of the BOPPPS-based blended teaching model in undergraduate nursing education: a quasi-experimental study. Sci Rep.

[24] Deci EL, Ryan RM (2000). The “what” and “why” of goal pursuits: human needs and the self-determination of behavior. Psychol Inq.

[25] Liu XY, Lu C, Zhu H (2022). Assessment of the effectiveness of BOPPPS-based hybrid teaching model in physiology education. BMC Med Educ.

[26] Alberti S, Motta P, Ferri P, Bonetti L (2021). The effectiveness of team-based learning in nursing education: a systematic review. Nurse Educ Today.

[27] Labrague LJ, Hammad K, Gloe DS (2018). Disaster preparedness among nurses: a systematic review of literature. Int Nurs Rev.

[28] Chen L, Tang XJ, Chen XK, Ke N, Liu Q (2022). Effect of the BOPPPS model combined with case-based learning versus lecture-based learning on ophthalmology education for five-year paediatric undergraduates in Southwest China. BMC Med Educ.

[29] Mee J, Pandian R, Wolczynski J (2024). An experimental comparison of multiple-choice and short-answer questions on a high-stakes test for medical students. Adv Health Sci Educ Theory Pract.

[30] Thijssen DHJ, Hopman MTE, van Wijngaarden MT, Hoenderop JGJ, Bindels RJM, Eijsvogels TMH (2019). The impact of feedback during formative testing on study behaviour and performance of (bio)medical students: a randomised controlled study. BMC Med Educ.

[31] Theobald KA, Tutticci N, Ramsbotham J, Johnston S (2021). Effectiveness of using simulation in the development of clinical reasoning in undergraduate nursing students: a systematic review. Nurse Educ Pract.

